# Overcoming bortezomib resistance in human B cells by anti-CD20/rituximab-mediated complement-dependent cytotoxicity and epoxyketone-based irreversible proteasome inhibitors

**DOI:** 10.1186/2162-3619-2-2

**Published:** 2013-01-10

**Authors:** Sue Ellen Verbrugge, Marjon Al, Yehuda G Assaraf, Denise Niewerth, Johan van Meerloo, Jacqueline Cloos, Michael van der Veer, George L Scheffer, Godefridus J Peters, Elena T Chan, Janet L Anderl, Christopher J Kirk, Sonja Zweegman, Ben AC Dijkmans, Willem F Lems, Rik J Scheper, Tanja D de Gruijl, Gerrit Jansen

**Affiliations:** 1 Department of Rheumatology, VU University Medical Center, Amsterdam, The Netherlands; 2 The Fred Wyszkowski Cancer Research Laboratory, Faculty of Biology, The Technion-Israel Institute of Technology, Haifa, Israel; 3 Department of Hematology, VU University Medical Center, Amsterdam, The Netherlands; 4 Department of Medical Oncology, VU University Medical Center, Amsterdam, The Netherlands; 5 Department of Pathology, VU University Medical Center, Amsterdam, The Netherlands; 6 Onyx Pharmaceuticals, South San Francisco, CA, USA

**Keywords:** Proteasome inhibitors, Anti-CD20/rituximab therapy, B cells, Autoimmune disorders, Resistance

## Abstract

**Background:**

In clinical and experimental settings, antibody-based anti-CD20/rituximab and small molecule proteasome inhibitor (PI) bortezomib (BTZ) treatment proved effective modalities for B cell depletion in lymphoproliferative disorders as well as autoimmune diseases. However, the chronic nature of these diseases requires either prolonged or re-treatment, often with acquired resistance as a consequence.

**Methods:**

Here we studied the molecular basis of acquired resistance to BTZ in JY human B lymphoblastic cells following prolonged exposure to this drug and examined possibilities to overcome resistance by next generation PIs and anti-CD20/rituximab-mediated complement-dependent cytotoxicity (CDC).

**Results:**

Characterization of BTZ-resistant JY/BTZ cells compared to parental JY/WT cells revealed the following features: (a) 10–12 fold resistance to BTZ associated with the acquisition of a mutation in the *PSMB5* gene (encoding the constitutive β5 proteasome subunit) introducing an amino acid substitution (Met45Ile) in the BTZ-binding pocket, (b) a significant 2–4 fold increase in the mRNA and protein levels of the constitutive β5 proteasome subunit along with unaltered immunoproteasome expression, (c) full sensitivity to the irreversible epoxyketone-based PIs carfilzomib and (to a lesser extent) the immunoproteasome inhibitor ONX 0914. Finally, in association with impaired ubiquitination and attenuated breakdown of CD20, JY/BTZ cells harbored a net 3-fold increase in CD20 cell surface expression, which was functionally implicated in conferring a significantly increased anti-CD20/rituximab-mediated CDC.

**Conclusions:**

These results demonstrate that acquired resistance to BTZ in B cells can be overcome by next generation PIs and by anti-CD20/rituximab-induced CDC, thereby paving the way for salvage therapy in BTZ-resistant disease.

## Background

Since the initial approval of rituximab, a monoclonal antibody against CD20, in 1997, it has become the cornerstone in the treatment of B cell lymphoproliferative diseases [1]. Additionally, B cells play a pivotal role in the pathogenesis of inflammatory autoimmune disorders such as rheumatoid arthritis (RA) and systemic lupus erythematosus (SLE), for which reason over the past decade rituximab has been added as B cell depleting therapy of auto-immune diseases [2-6]. However, it is well recognized that a considerable number of patients are either primary resistant to rituximab, or experience a limited period of response [5,7]. The efficacy of rituximab is associated with proficient CD20 expression on the surface of B cells, and indeed, acquisition of rituximab resistance appears to be correlated with down-regulation of CD20 [8,9]. Therefore, there is an urgent need for new therapeutic modalities. Beyond immunotherapy approaches, small molecule targeting of B-cells has been achieved with proteasome inhibitors (PIs) such as bortezomib (BTZ) [10]. Bortezomib-induced apoptosis and cell death has been reported for a large panel of human B cell lines, including those resistant to rituximab therapy [11-13]. Accordingly, a clinical effect of BTZ in B cell driven diseases has been found. BTZ is highly effective in inducing plasma cell death, thus, is now being implemented both in first line and relapsed and refractory multiple myeloma (MM) [14]. BTZ has also shown clinical efficacy in Mantle Cell Lymphoma [15] and Waldenström’s Macroglobulinemia [16], the latter also in combination with rituximab [17], indicating its effectivity in inducing cell death in earlier stages of B-cell development. In the autoimmune disease setting, clinical studies with PIs are far less advanced, although in pre-clinical studies, PI therapy demonstrated promising results as anti-arthritic treatment strategy, also by B cell depletion [18-22].

One hypothesis for proficient B cell targeting by PIs is that their substantial turnover and secretion of proteins renders these cells highly susceptible to interference in protein homeostasis, in particular following inhibition of the ubiquitin proteasome system (UPS) [23]. The UPS is the master regulator of cellular homeostasis by mediating ubiquitination and degradation of proteins that control apoptosis induction, and also mediate pro-inflammatory cytokine production via NFκB activation [24-26]. Most mammalian cells express constitutive proteasomes which harbor the β5 subunit (PSMB5), the β1 subunit (PSMB6) and the β2 subunit (PSMB7) and cleave proteins (or long peptides) after hydrophobic, acidic, and basic amino acid residues, respectively [27,28]. After stimulation with IFN-γ and/or TNF-α, these constitutive proteasome subunits are replaced by immunoproteasome subunits: β5i (LMP7), β1i (LMP2), and β2i (MECL1) [29]. These proteasome entities are part of the immunoproteasome, which is mainly expressed in cells of hematological origin and can generate a distinct peptide repertoire for MHC-class I presentation [30]. As a first generation PI, Bortezomib targets both constitutive and/or immunoproteasome subunits via a reversible binding to the proteasome, however, nowadays several next-generation PIs have been rationally designed and tested, harboring the capacity to specifically target the constitutive and/or immunoproteasome subunits through irreversible binding [23,28,29,31-35].

Given the chronic nature of autoimmune-related diseases, with patients facing long-term treatment, it is anticipated that PI treatment may also be accompanied by loss of treatment efficacy/onset of drug resistance over time (Kumar *et al.*, 2012), as has been described in extensively-treated MM patients [36,37]. We, as well as others, have described the development of resistance to BTZ in several models of hematological cancer cells, including T-cells, myeloid cells, and plasma cells [38-41]. For both benign and neoplastic B cells, however, the long term effects of BTZ exposure are largely unexplored and deserve further investigation.

In this study, we investigated the long-term effects of B-cell exposure to BTZ and onset of resistance to BTZ using EBV-transformed JY lymphoblast cells as a benign autoimmune disorder *in vitro* B-cell model. Beyond characterizing the molecular basis of resistance to BTZ in JY cells, we report two modalities to overcome BTZ resistance in B cells; (a) by next-generation epoxyketone-based irreversible PIs, and (b) by enhanced rituximab-mediated complement-dependent cytotoxicity (CDC), exploiting the upregulated cell surface expression of CD20 in BTZ-resistant JY cells.

## Results

### Acquisition of bortezomib resistance and cross-resistance to other proteasome inhibitors

The human JY B cell line was used as a model to examine the cellular effects of long-term exposure of B cells to BTZ, including the acquisition of BTZ resistance. Cell cultures were exposed to a low concentration of BTZ (IC_10_: 1.5 nM) which was gradually (stepwise) increased to 100 nM of BTZ over a period of 6 months. JY cells stably growing in BTZ concentrations of 35nM (JY/35) and 100nM (JY/100) were used for further characterization.

**Figure 1:**
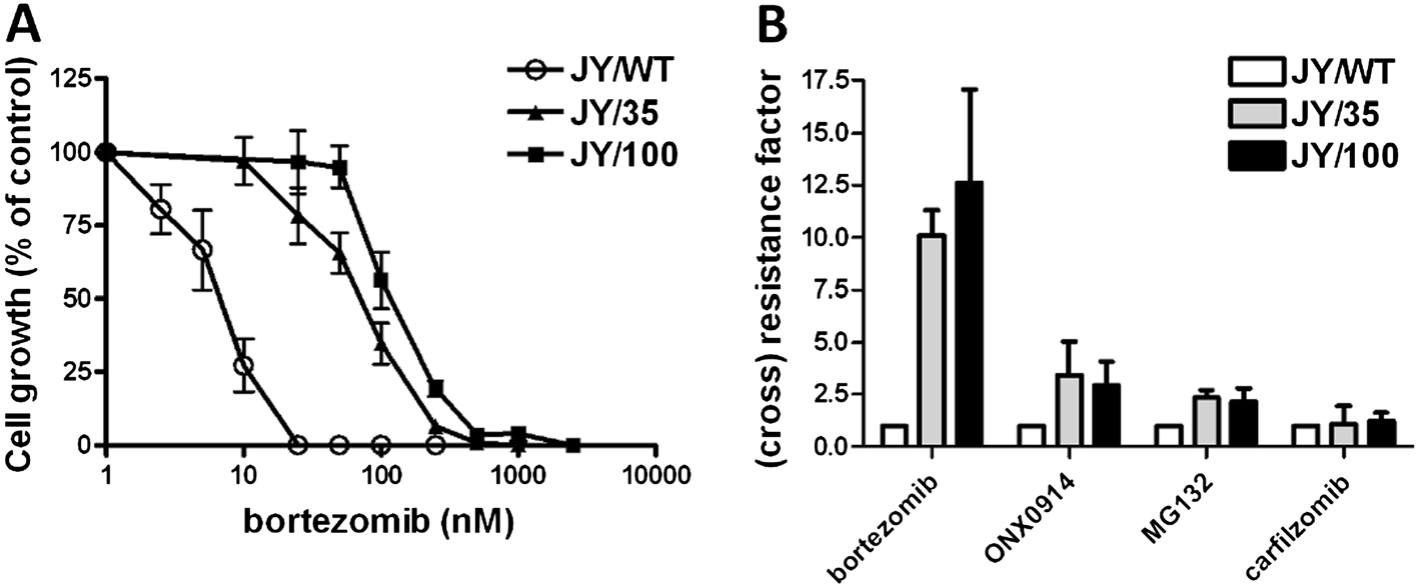
**Acquisition of bortezomib (BTZ) resistant JY cells and proteasome inhibitor cross-resistance profile. (A)** Dose–response curve for BTZ-induced growth inhibition of JY/WT and BTZ-resistant variants JY/35BTZ and JY/100BTZ following 72 hours exposure to BTZ. Results represent the mean ± SD of 6 separate experiments. **(B)** Resistance factor for BTZ and cross- resistance factors to ONX 0914 (immunoproteasome inhibitor), MG-132, and carfilzomib. Cell growth inhibition was assessed after 72 hours incubation with PIs. (Cross) resistance factor represents the ratio of drug concentration required to inhibit cell growth of BTZ-resistant JY cells by 50% over JY/WT cells. Results are the mean ± SD of 3–5 separate experiments.

Figure 1A shows the growth inhibition profiles of JY/WT, JY/35 and JY/100 cells after 72 hrs treatment with BTZ, revealing a clear shift in drug concentrations causing 50% cell growth inhibition (IC_50_ value) in JY/35 and JY/100 cells. Figure 1B shows a 10-fold and 12-fold BTZ resistance in JY/35 and JY/100 cells, respectively, compared to JY/WT cells. Cross-resistance to other proteasome inhibitors (PI) was also determined in JY/35 and JY/100 cells; a low level of cross-resistance was found for the specific immunoproteasome inhibitor ONX 0914 (resistance factor: 3.4 and 2.9, respectively) and the pan-proteasome inhibitor MG-132 (resistance factor: 2.3 and 2.2, respectively). Remarkably, however, JY/35 and JY/100 cells retained full sensitivity towards the next-generation irreversible PI, carfilzomib.

### Molecular alterations in BTZ-resistant JY cells

**Figure 2:**
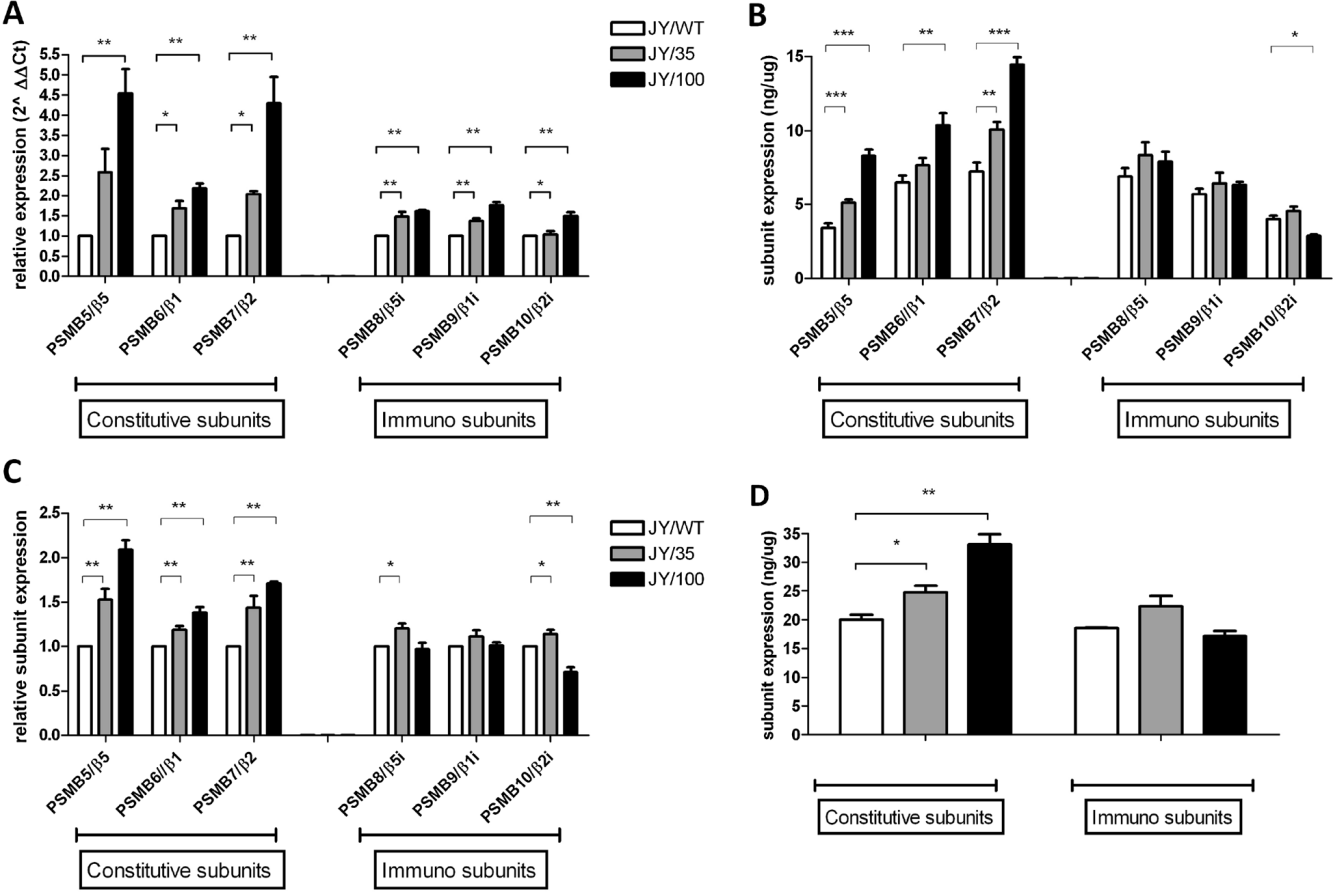
**Constitutive and immunoproteasome composition in JY cells and BTZ-resistant variants. (A)** Relative mRNA levels of constitutive and immunoproteasome subunits in JY/WT and BTZ-resistant variants JY/35BTZ and JY/100BTZ. mRNA levels were normalized relative to β-glucuronidase as house keeping gene. **(B)** Protein expression of constitutive and immunoproteasome subunits in JY/WT and BTZ-resistant variants JY/35BTZ and JY/100BTZ. **(C)** Relative protein expression of constitutive and immunoproteasome subunits in JY/WT and BTZ-resistant variants JY/35BTZ and JY/100BTZ. **(D)** Total constitutive and immunoproteasome subunit expression in JY/WT and BTZ-resistant variants JY/35BTZ and JY/100BTZ. Results depicted are means ± SD of 3–5 separate experiments. *** p < 0.001, ** p < 0.01, * p < 0.05.

We and others [38-41] have previously discovered a mechanism of BTZ resistance in various haematological cell lines. This mechanism included BTZ-induced single point mutations in the PSMB5 gene, as well as a selective overexpression of mutant PSMB5 protein [38,39,41]. Here, we tested whether this mechanism would also apply for BTZ-resistant JY cells. We first determined whether *PSMB5* harbored any of the previously described mutations [38-41]. Indeed, in JY/35 cells as well as JY/100 cells, a single G to T nucleotide shift was identified at nucleotide position 311 in exon 2 of the *PSMB5* gene (Additional file 1: Figure S1). In the mature and functional β5-subunit protein, this mutation introduced a Met to Ile substitution at amino acid 45. It is noteworthy that the mutation in *PSMB5* in JY/35 and JY/100 cells is heterozygous, indicating that these cells would still harbor one unaffected allele. We next determined the expression of the constitutive and immunoproteasome subunits at both mRNA and protein levels (Figure 2). Compared to JY/WT cells, mRNA levels of the constitutive proteasome subunits, in particular PSMB5 (β5) and PSMB7 (β2), were significantly increased (up to 4.5 fold) in JY/BTZ cells, whereas the increase in the mRNA of immunoproteasome subunits was much less pronounced (Figure 2A). Consistent with these data, protein levels of constitutive proteasome subunits were increased in BTZ-resistant JY cells (up to 2-fold), whereas the expression levels of immunoproteasome subunits remained largely unaltered (Figure 2B-D).

### Surface molecules involved in antigen-presentation

JY cells are efficient antigen-presenting cells [42] and we therefore examined whether or not BTZ selection would induce alterations in markers involved in antigen presentation. In fact, no significant changes were found in the cell surface expression of HLA-ABC (MHC-class I), HLA-DR (MHC-class II), and the co-stimulatory molecules CD80, CD86, and CD40 in JY/35 and JY/100 cells compared to JY/WT cells (Additional file 1: Figure S2). Further confirmation that BTZ selection had no impact on antigen presentation was provided by the observation that JY/WT and BTZ-resistant cells showed no difference in their capacity to induce allogeneic T-cell proliferation (Additional file 1: Figure S3). Of note, short term (24 hr) incubations with maximal non-toxic concentrations of BTZ also did not affect their ability to induce proliferation of allo-T-cells (Additional file 1: Figure S3).

### Cytokine release capacity by BTZ-resistant JY cells

**Figure 3:**
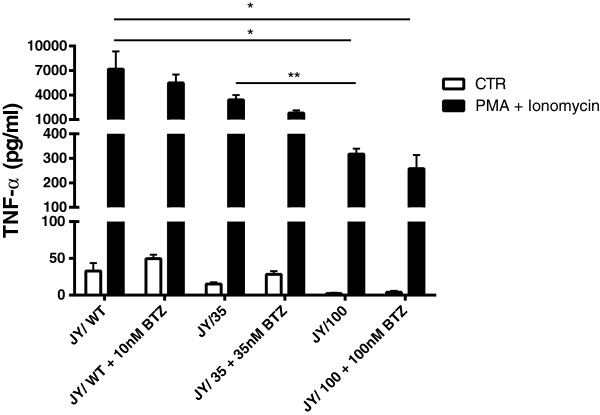
**TNF-α release by JY/WT cells and BTZ-resistant variants JY/35BTZ and JY/100BTZ.** Cells were activated for 24 hours with 10 ng/ml PMA and 750 ng/ml Ionomycin, in the absence or presence of the indicated concentration bortezomib. Results depicted are means ± SD of 3 separate experiments. ** p < 0.01, * p < 0.05.

Activated B cells are able to produce cytokines and consequently contribute to inflammation [43] or protective cytokine environments [44]. To this end, we examined the secretion profile of the prototypical pro-inflammatory cytokine TNF-α by activated JY cells and explored whether or not this profile is altered in JY/BTZ cells. Beyond basal secretion levels, we also assessed the impact of co-incubation with BTZ on TNF-α production. Upon activation with PMA and ionomycin, JY cells produced TNF-α levels of 7000 ± 3700 pg/ml, which was diminished by 20% in the presence of 10 nM BTZ (Figure 3). Basal levels of TNF-α production in JY/35 cells was approximately half of JY/WT cells, and could be further suppressed in the presence of 35 nM BTZ. Strikingly, basal TNF-α production in JY/100 was markedly decreased to levels < 5% of JY/WT cells. Together, these results indicate that upon acquisition of BTZ resistance, JY cells are markedly suppressed in their capacity to secrete TNF-α*.*

### CD19 and CD20 expression in BTZ-resistant JY cells

**Figure 4:**
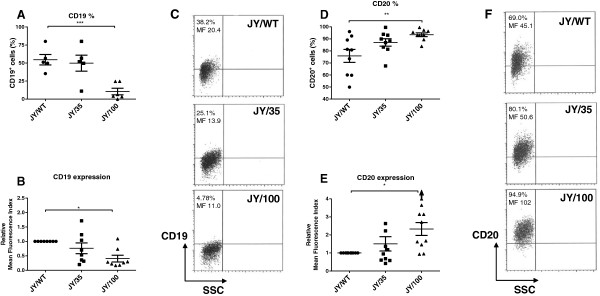
**Expression of B cell surface markers CD19 and CD20 on JY/WT cells and BTZ-resistant variants. (A)** Percentage CD19-positive JY/WT cells and BTZ-resistant variants. **(B)** Expression of CD19 in BTZ-resistant JY cells relative to JY/WT cells. Results are depicted as relative Mean Fluorescence Index (MFI) and represent the mean ± SEM of 5 separate experiments. **(C)** Representative figure of CD19 expression in JY/WT cells and BTZ-resistant variants. Percentages and mean fluorescence intensity are indicated in the upper left quadrant. **(D)** Percentage CD20-positive JY/WT cells and BTZ-resistant variants. **(E)** Expression of CD20 in BTZ-resistant JY cells relative to JY/WT cells. Results depicted are depicted as relative Mean Fluorescence Index (MFI) and represent the mean ± SEM of 9 separate experiments. **(F)** Representative figure of CD20 expression in JY/WT cells and BTZ-resistant variants. Percentages and mean fluorescence intensity are indicated in the upper left quadrant. *** p < 0.001, ** p < 0.01, * p < 0.05.

CD19 and CD20 are prototypical B cell surface markers, and lost upon differentiation to plasma cells [2,3,45]. We examined whether or not induction of BTZ-resistance altered CD19 and/or CD20 expression levels in JY/BTZ cells. Flow cytometric analyses revealed a significant decrease in CD19+ cells (55 ± 16%) in JY/WT cells to 14 ± 11% in JY/100 cells (Figure 4A) along with a concomitant 3-fold decrease in CD19 cell surface expression (Figure 4B). Interestingly, CD20 expression profiling was the inverse of CD19 in positive cells (Figure 4C), whereas CD20 cell surface expression was 2.8-fold higher in the resistant JY/100 cells than JY/WT cells (Figure 4D).

### Regulation of CD20 expression in BTZ-resistant JY cells

**Figure 5:**
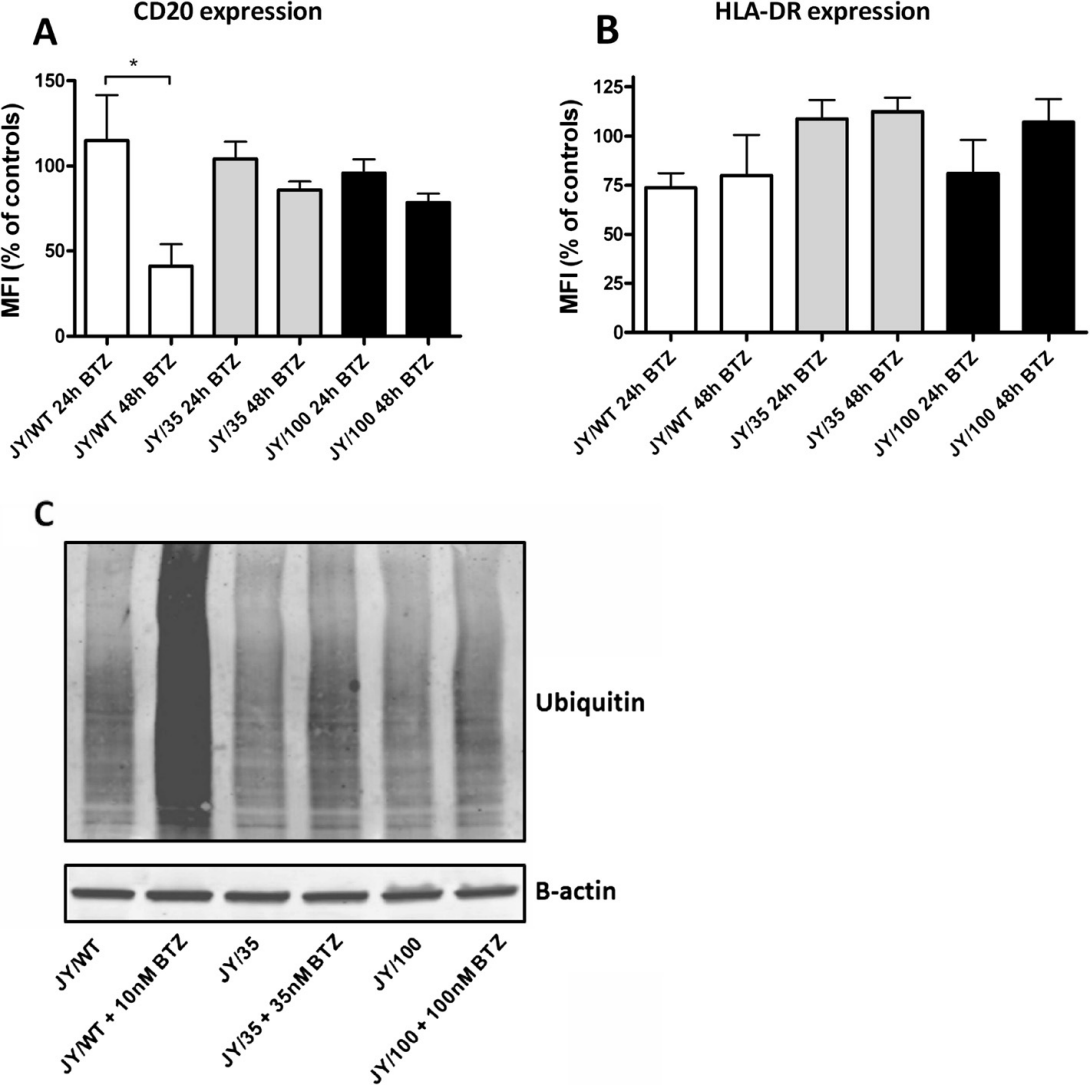
**Effect of BTZ on CD20 (A) and HLA-DR (B) expression in JY/WT and BTZ-resistant JY cells.** Short term incubations of 24 hours and 48 hours were performed with 5nM BTZ for JY/WT, 35nM for JY/35BTZ cells and 100nM BTZ for JY/100BTZ cells. Results depicted are means ± SD of 3–4 separate experiments. * p < 0.05. **(C)** Western blot analysis of polyubiquitinated cellular proteins prior to and after 24 hours treatment of BTZ; JY/WT (± 10nM BTZ), JY/35 (± 35nM BTZ) and JY/100 (± 100nM BTZ). β-Actin is displayed as a loading control. Western blot image is representative of 3 separate experiments.

Recently, it has been reported [46] that proteasome inhibition and ubiquitination of CD20 triggers its degradation and decreases its cell surface expression. Consistent with this notion, we observed that CD20 expression was significantly decreased on JY/WT cells exposed for up to 48 hrs to 5 nM BTZ (Figure 5A). In comparison, no effect of BTZ treatment was observed for HLA-DR expression (Figure 5B). Unlike in JY/WT cells, treatment of JY/35 and JY/100 cells with BTZ (35 nM and 100 nM BTZ, respectively) had no significant effect on CD20 expression (Figure 5A). In order to establish whether protein ubiquitination status after short-term (24–48 hr) BTZ treatment is associated with down-regulation of CD20 expression in JY/WT cells and unaltered CD20 expression in JY/BTZ cells, Western blot analysis of total ubiquitinated proteins was performed with control and BTZ- exposed cells. Figure 5C shows a typical smear of (poly) ubiquitinated proteins when JY/WT cells were incubated with 10 nM BTZ for 24 hrs. Conversely, no accumulation of ubiquitinated proteins were observed in JY/35 and JY/100 cells exposed to 35 nM and 100 nM BTZ, respectively. These results suggest that aberrant protein ubiquitination and degradation could be a contributing factor towards elevated levels of CD20 in BTZ-resistant JY cells.

### Increased sensitivity to anti-CD20/rituximab-mediated CDC in bortezomib-resistant JY cells

**Figure 6:**
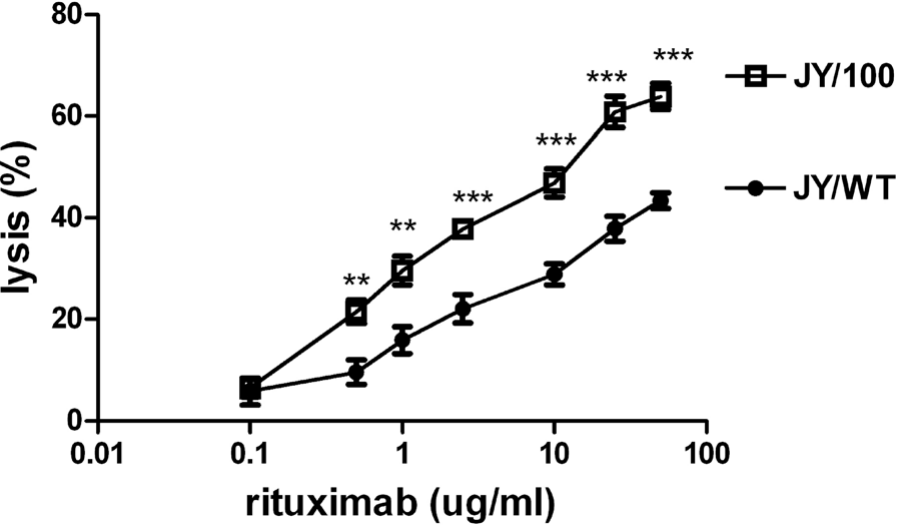
**Rituximab-induced CDC in JY/WT and BTZ-resistant JY cells.** Cells were incubated with increasing concentrations anti-CD20/rituximab (range; 0.1 μg-50 μg/ml) and 5% baby rabbit complement serum. Cell viability (% lysis) was measured after 1 hour using propidium iodide (PI) staining and FACS analysis. Results depicted are means ± SD of 5–8 separate experiments. *** p < 0.001, ** p < 0.01.

To explore whether or not increased expression levels of CD20 in JY/100 cells is therapeutically relevant, we investigated its impact on anti-CD20/rituximab-induced complement-dependent cytotoxicity (CDC). In the presence of 5% baby rabbit serum [47] and over a broad range of rituximab concentrations, significantly increased CDC was noted for JY/100 cells as compared to JY/WT cells with a mean difference of 17% lysis (Figure 6). This increase was not associated with alterations in CD55 and/or CD59 expression levels between JY/WT and JY/BTZ cells, two markers implicated in controlling CDC [9,46] (not shown). Together, these results suggest that increased expression levels of CD20 in JY/BTZ cells may be exploited as an alternative therapy to combat BTZ resistance through rituximab-induced CDC.

## Discussion

The current study describes two ways to overcome acquired resistance to BTZ in JY human B cells: (a) anti-CD20/rituximab-mediated CDC, and (b) next generation epoxyketone-based irreversible PIs.

B cell depleting therapies utilizing the anti-CD20/rituximab antibody approach, as well as the small molecule approach with the proteasome inhibitor BTZ, have demonstrated clinical efficacy in the treatment of lymphoproliferative disorders [1,10,23,48]. In the treatment of autoimmune diseases, rituximab also now has an accepted place [3-5,43], but BTZ or other PIs still await clinical evaluation. Ideally, based on their non-overlapping mechanisms of action, rituximab and bortezomib could potentially replace each other when development of resistance to one or the other occurred. In this context, it has been reported that rituximab-resistant B cells retain sensitivity for BTZ, but the reverse has not been demonstrated. In view of the need for chronic treatments in autoimmune diseases, there are concerns about the emergence of resistance [36], thus highlighting the need to unravel underlying mechanisms of resistance, in order to design strategies to overcome acquired resistance. To address these issues, we provoked acquired resistance to BTZ in a human B cell line model and investigated its impact on rituximab sensitivity and next-generation PIs designed to overcome BTZ-resistance.

Here we mimicked the onset of acquired BTZ resistance in human B cells using human JY lymphoblastic cells as benign autoimmune disorder *in vitro* model, by a classical approach of cell exposure to stepwise increasing concentrations of BTZ (5 through 100 nM), representing clinically-achievable plasma levels [10,23]. Characterization of the BTZ-resistant JY cells (10–12 fold compared to WT cells) revealed the following main features: 1) acquisition of a point mutation in the *PSMB5* gene (encoding for the constitutive β5 proteasome subunit) along with overexpression of β5 subunit protein, 2) retention of sensitivity to epoxyketone-based PIs including carfilzomib, 3) an altered cytokine environment indicated by a marked reduction in the secretion of the inflammatory cytokine TNF-α, and 4) immunophenotypically, a significantly reduced expression of the B cell surface marker CD19 and a concomitant increase in CD20 levels.

BTZ-resistance associated with a single point mutation in *PSMB5* has been demonstrated by us and others for leukemia cell lines of lymphoid and myeloid origins, as well as solid tumor cell lines [38-41,49]. These mutations introduced amino acid alterations that clustered in critical positions (Thr21, Met45, Ala49 and Cys52) in the BTZ binding pocket within the β5 subunit [41]. Based on data from the present study, it is revealing that beyond hematological malignant cells, *PSMB5* mutations can also be identified in BTZ-resistant JY lymphoblastic cells. It is noteworthy that the mutation in *PSMB5* in JY/BTZ cells is heterozygous, indicating that cells still retained an intact WT allele. This may underlie a relatively lower level of BTZ resistance observed in JY/BTZ cells as compared to the substantially higher resistance levels (> 100-fold) noted in leukemic cells harboring homozygous mutations in *PSMB5*[38,41]. Notwithstanding this fact, expression levels of mutant β5 subunits were upregulated in JY/BTZ cells, likely as a compensatory mechanism to sustain basal proteasome activity. The Met45Ile substitution identified in BTZ-resistant JY cells consistently resided in the highly-conserved substrate/inhibitor-binding domain of the β5 subunit. Met45 is well recognized to be essential for proteasome-substrate interactions, as well as for binding of the leucine boronic acid moiety (P1 site) of BTZ [29,32,50]. In this context, it may be anticipated that replacement of the non-polar, spatially flexible Met45 for another slightly more non-polar, bulkier, and more rigid amino acid as Ile, would affect optimal BTZ binding, thereby resulting in the conferring of drug resistance. From this perspective, it is intriguing to note that the Ala49 and Met45 mutations in the β5 subunit were also identified in marine bacteria as a self-protection mechanism against the natural PI, salinosporamide A, that they produce [51].

A major finding in the current study is that JY/BTZ cells harboring the Met45Ile mutation in the β5 subunit retained full sensitivity to carfilzomib, an epoxyketone-based PI. Previous studies from our laboratory also indicated that carfilzomib retained appreciable activity against BTZ-resistant leukemia [41,52] and solid tumor cell lines [23,35,49]; similar findings were observed with ONX 0912 [53], an orally- bioavailable analog of carfilzomib which provokes irreversible binding via the epoxyketone group to the active site Thr1 residue in the β5 subunit. This unique property, rather than reversible binding by boronate-based BTZ, may still facilitate inhibition of the catalytic activity in mutated β5, thereby resulting in retention of drug sensitivity. Since levels of immunoproteasome subunits were largely unaltered and no mutations were observed in the β5i subunit in JY/BTZ cells, it may also explain the marginal levels of cross-resistance that were observed for the immunoproteasome-specific inhibitor ONX 0914. In this context, it is worthwhile mentioning that recent crystal structure studies identified Met45 in β5i as a major determinant in the efficient docking of ONX 0914 to the catalytic binding site [29]. The Met45Ile mutation in the constitutive β5 subunit has apparently lesser consequences for ONX 0914 activity [20]. Together, constitutive and immunoproteasome targeted epoxyketone-based PIs like carfilzomib, currently evaluated in phase I-III clinical trials [54], as well as ONX 0914 may readily overcome BTZ-resistance.

Besides antiproliferative effects, BTZ can also elicit potential anti-inflammatory effects by suppression of the release of pro-inflammatory cytokines through inhibition of NF-κB activation [18-20,25,26]. In this respect, TNF-α has received the most attention as one of the critical cytokines in the pathophysiology of arthritis, and is thus a major druggable target for biological agents and small molecules [20-22,48]. Consistent with data from the Komano group [55], we noted that JY/WT cells could produce appreciable levels of TNF-α, which could be partially inhibited by BTZ. Of additional interest was the notion that alongside these short term effects, acquisition of BTZ resistance in JY cells provoked a dramatic reduction in the basal levels of TNF-α production, being most pronounced in JY/100 cells. It remains to be established whether or not this effect is due to diminished response to activation stimuli, or aberrations in TNF-α secretion *per se*. In either case, prolonged BTZ exposure may elicit potential anti-inflammatory effects even when anti-proliferative effects are compromised.

Acquisition of BTZ resistance in JY cells was associated with opposite impacts on cell surface expression of two common B-cell surface markers: down regulation of CD19 and upregulation of CD20. CD19 is a transmembrane protein and co-receptor molecule that modulates signals through the B cell antigen receptor (BCR) to control B cell-differentiation from pro/pre-B cells to resting pre-B cells in bone marrow. Consistently, CD19^−/−^ mice have exhibited defects at stages of early B cell development and reduced levels of circulating B-cells, as reviewed in Del Nagro et al. [45]. It is yet unclear whether reduced CD19 levels in JY/BTZ cells would result in a B cell differentiation arrest; this should be subject for further studies using panels of B cell differentiation-restricted cell surface markers.

CD20 serves as a Ca^2+^-permeable cation channel protein expressed on B cells from immature to mature stages of differentiation, as recently reviewed by Thaunat et al. [2]. BTZ has been reported to induce disregulation of intracellular Ca^2+^, triggering caspase activation and initiating apoptosis [56]. It is unknown whether this off-target effect of BTZ alters CD20 function and expression during resistance development. Rather, recent studies have pointed to the role of the ubiquitin proteasome system in the regulation of CD20 expression, as rituximab-resistant lymphoma cells have displayed upregulated expression of proteasome subunits [8], and treating Raji human Burkitt lymphoma cells with BTZ for 24–48 hours has down-regulated CD20 expression [46]. In accordance with these studies, we now showed that impaired (poly) ubiquitination of proteins upon 24–48 hour BTZ treatment in resistant JY/BTZ cells attenuated degradation of CD20, resulting in a net 3-fold increase in CD20 expression in JY/100 cells and markedly enhanced rituximab-mediated CDC in JY/100 cells as compared to JY/WT cells. Based on the differential impact on CD20 expression in BTZ-sensitive and BTZ-resistant cells, one should take caution when considering combining rituximab and BTZ for B cell targeting; efficacy may only be realized in BTZ-resistant cells. Moreover, the current findings for JY lymphoblastic cells should also await further corroboration for B-cell neoplasms (e.g. mantle cell lymphoma) for which BTZ is a therapeutic option. Finally, it would be of additional future interest to explore whether aberrations in protein ubiquitination provoked by prolonged exposure to either BTZ or other types of PIs, may be instrumental to upregulated expression of other B cell surface markers that could elicit antibody-mediated CDC.

## Conclusions

In conclusion, this study presented two novel strategies to overcome acquired resistance to BTZ in human B lymphoblastic cells: one is a small molecule approach with irreversible epoxyketone-based PIs, whereas the second relates to an immunological approach by anti-CD20/rituximab-mediated CDC, exploiting the upregulation of CD20 in BTZ-resistant cells. This deserves further exploration in order to improve the clinical outcome in patients with bortezomib-resistant diseases.

## Methods

### Cell culture and development of BTZ-resistant cell lines

The human EBV-transformed B-lymphoblastic cell line JY (ATCC) was cultured in IMDM-medium (PAA, The Cell Culture Company, PAA Laboratories GmbH, Germany) supplemented with 10% FCS, 1% β-mercaptoethanol, 1% penicillin, 1% streptomycin and 2 mM L-glutamine. BTZ-resistant JY cell lines were established by stepwise increasing concentrations of BTZ over a period of 6 months, starting at a BTZ concentration of 1.5 nM (IC_10_ concentration; establishing 10% cell growth inhibition compared to the untreated control) up to a concentration of 100 nM. Cells stably growing in the presence of 35 nM (JY/35) and 100 nM BTZ (JY/100) were isolated after 3 months and 6 months of stepwise selection, respectively. Unless otherwise indicated, experiments were performed with cells cultured in drug-free medium for at least 4 days.

### Reagents / chemicals / antibodies

Bortezomib (BTZ)/Velcade™ was provided by Millennium Pharmaceuticals (Cambridge, USA). Carfilzomib and ONX 0914 were generously provided by Onyx Pharmaceuticals, Inc. (South San Francisco, USA). MG132 (Z-Leu-Leu-Leucinal) was purchased from Calbiochem/ Merck (Nottingham, UK). Phorbol-12-myristate 13-acetate (PMA) and ionomycin were from Sigma (St. Louis, MO, USA).

Rituximab, a chimeric immunoglobulin G1, was purchased from Roche (Roche Netherlands B.V., Woerden, The Netherlands). Other antibodies were from the following sources: ubiquitin: (P4D1, Santa Cruz Biotechnology, sc-8017) and β-actin (C4, Santa Cruz, sc-47778) mouse monoclonal antibodies; goat-anti-mouse secondary antibody conjugated to IRDye® 800CW (Odyssey; L1-COR Biosciences, Nebraska, USA).

### Isolation of mRNA, cDNA synthesis, and quantitative RT-PCR

Following RNA isolation with the RNeasy Mini kit (Qiagen), cDNA was synthesized using 0.75U/ul M-MLV reverse transcriptase (Invitrogen) in a RT buffer (Invitrogen), containing 5 mM DTT (Invitrogen), 2 mM dNTP (Roche), pdN6 96ug/ml (Roche), and 2U/ulRNAsin (HT Biotechnology Ltd., Cambridge, UK). mRNA expression levels of proteasome subunits PSMB5 (β5), PSMB6 (β1), PSMB7 (β2), PSMB8 (ß5i)*,* PSMB9 (ß1i)*,* PSMB10 (ß2i), and the endogenous housekeeping gene β-glucuronidase (GUS) as a reference, were quantified using real-time PCR analysis (Taqman) using an ABI Prism 7700 sequence detection system (PE Applied Biosystems, Nieuwerkerk a/d IJssel, The Netherlands) as described previously [41]. Briefly, for PSMB5, a Taqman gene expression assay was used according to the manufacturers’ instructions (Hs00605652_m1, Applied Biosystems). All other primers and probes were designed using Primer Express software (Applied Biosystems) and are indicated in Additional file 1: Table S1. Probes were labeled with 5’-FAM and 3’-BHQ1 as a reporter. Real-time PCR was performed in a total reaction volume of 25 μl containing TaqMan buffer A (Applied Biosystems), 4 mM MgCl_2_, 0.25 mM of each dNTP (Amersham Pharmacia Biotech), and 1.25 U AmpliTaq Gold DNA polymerase (Applied Biosystems). Samples were heated for 10 min at 95°C to activate the AmpliTaq Gold DNA polymerase and amplified during 40 cycles of 15 s at 95°C and 60 s at 60°C. Relative mRNA expression levels of the target genes in each sample were calculated using the comparative cycle time (Ct) method. The Ct of the target gene was normalized to the GUS Ct value by subtracting the GUS Ct value from the target Ct value. The mRNA expression level for each target PCR relative to GUS was calculated using the following equation: mRNA expression = 2(Ct target-Ct GUS) × 100%.

### Proteasome constitutive-immuno subunit ELISA assay (ProCISE)

The ProCISE assay for quantification of individual constitutive (β5, β1, β2) and immunoproteasome (β5i, β1i, β2i) subunits was performed as previously described [33,34]. Expression of each c20S and i20S subunit in JY/WT, JY/35, and JY/100 cells was examined after cells were cultured in the absence of BTZ for one week. Pellets of 10 x 10^6^ -15 x 10^6^ cells were washed three times with PBS, frozen on dry ice, thawed, then incubated in 100 μL of lysis buffer (20 mM Tris–HCl, pH 8.0, 5 mM EDTA) for 15 minutes on ice. Lysed pellets were micro-centrifuged (15 min, 14,000 rpm, 4°C), the supernatant collected, and protein content determined by BCA assay (Pierce), utilizing a BSA standard. Cell lysates were diluted in lysis buffer to 1 mg/mL, and then incubated with a proteasome active site probe (PABP; 5 μM) for 2 hours at 25°C. Samples were denatured with 8 M guanidine hydrochloride (Fisher Scientific) and subunits bound to PABP were captured with streptavidin-conjugated sepharose beads (GE Healthcare) in 96-well 0.65 μm porous filter plates (Millipore). Individual subunits were probed with subunit-specific primary antibodies, followed by secondary antibodies conjugated to horseradish peroxidase (HRP). The SuperSignal ELISA Pico chemiluminescent substrate kit (Pierce) was utilized to generate luminescent signal associated with HRP binding. Luminescence after 5 minutes of signal development was monitored on a plate reader (Tecan Safire). Subunit content was expressed as nanograms of subunit per microgram of total protein, as calculated from a standard curve of human purified constitutive or immunoproteasome, assayed simultaneously with the cell lysate samples.

### Cytokine release assay

JY cells were seeded in 96-wells plates at a cell density of 5 x 10^4^/200μl and stimulated with 10 ng/ml PMA (Sigma) and 750 ng/ml ionomycin (Sigma). After 24 hours, culture supernatants were collected and analyzed using the cytometric bead array (CBA) human inflammatory cytokines kit (BD Biosciences, San Jose, CA) according to the manufacturer’s protocol. Quantitative measurements of TNF-α were performed using a FACSCalibur flow cytometer (Becton and Dickinson, San Jose, CA) with the CellQuest program.

### FACS analysis – immunophenotyping

JY cells were immunophenotyped using FITC-, PE- and/or APC-conjugated monoclonal antibodies (Mabs) directed against: CD80 (1:10), CD86 (1:10), CD40 (1:10) HLA-ABC (1:25), HLA-DR (1:25), CD19 (1:10), and CD20 (1:10) (BD Biosciences, San Jose, CA) and CD54 (1:10) (Leinco Technologies Inc., St. Louis, USA). In short, 3 x 10^4^ – 8 x 10^4^ cells were washed with PBS supplemented with 0.1% BSA and 0.02% NaN_3_, and incubated with specific or corresponding isotype-matched control Mabs for 30 minutes at 4°C. Cells were washed and analyzed with a FACSCalibur flow cytometer (Becton and Dickinson, San Jose, CA) using the Cell Quest program. Results were expressed as percentages of positive cells and the mean fluorescence index was calculated based on mean fluorescence intensities.

### Western blot analysis

Accumulation of ubiquitinated proteins upon treatment with BTZ was determined by Western blot analysis as described previously [38]. In short, 3 × 10^6^ JY/WT, JY/35 and JY/100 cells were incubated in 10 ml growth medium in the absence or presence of 10nM, 35nM and 100nM BTZ, respectively. After 24 hours of incubation, cells were harvested and washed 3 times with ice-cold PBS at pH 7.4. Total cell lysates were prepared by resuspending in 0.1 ml lysis buffer (Cell Signaling Technology) containing: 20% PIC (Protease Inhibitor Cocktail) and 10% NaVO_4_. The suspension was sonicated (MSE sonicator, amplitude 7, for 3 × 5 seconds with 20-second time intervals at 4°C) and centrifuged in an Eppendorf micro-centrifuge (5 min, 12,000 rpm, 4°C). Protein content of the supernatant was determined by the Bio-Rad Protein Assay using a bovine serum albumin (BSA) standard. Protein aliquots (30 μg) of total cell lysates were fractionated on a 4-20% TGX pre-cast gel (BioRad) containing SDS, and transferred onto a PVDF membrane. The membranes were pre-incubated for 1 hour in blocking buffer (Rockland Immunochemicals Inc., Gilbertsville, PA, USA) to prevent non-specific antibody binding. After blocking, the membranes were incubated for 1 hour at room temperature with an ubiquitin-specific antibody (1:200). After 3 washing steps with Tris-buffered saline with 0.5% Tween, the membranes were incubated for 1 hour with goat-anti-mouse secondary antibody conjugated to IRDye ® 800CW (1:10000, Odyssey; L1-CORBiosciences, Nebraska, USA). Detection of antibody binding was obtained using the LI-COR Odyssey scanner (Biosciences) according to the manufacturers’ instructions, and digital image acquisition and quantification was performed using the Odyssey infrared imaging system software (version 3.0.16, LI-COR Biosciences, Nebraska, USA). Expression of ubiquitinated proteins was detected using a mouse monoclonal antibody (1:200) as described above. β-actin (1:1000 diluted antibody) was used as the control for equal loading.

### Complement-dependent cytotoxicity (R-CDC) assay

JY cells (5 x 10^4^cells) were plated in triplicate in 96-well plates in IMDM medium with or without rituximab (concentrations ranging from 0.1 μg-50 μg/ml) and 5% baby rabbit complement serum (Cedarlane, Ontario, Canada). After 1 hour of incubation at 37°C, the numbers of non-viable cells were determined after propidium iodide (PI) staining using flow cytometry. The percentage of lysed (PI+) cells was calculated based on a total of 1.2 x 10^4^ measured events. Cell lysis in control conditions (without baby rabbit serum) was subtracted to obtain specific complement-dependent lysis.

### Statistical analysis

For comparisons between groups, ANOVA and Student’s t-test were used. Differences were considered to be significant at p < 0.05.

## Abbreviations

BTZ: Bortezomib; TNF-α: Tumor Necrosis Factor- α; PI: Proteasome inhibitor; CDC: Complement-dependent cytotoxicity.

## Competing interests

SEV: no competing interests to declare; MA: no competing interests to declare; YGA: no competing interests to declare; DN: no competing interests to declare; JM: no competing interests to declare; JC: no competing interests to declare; MV: no competing interests to declare; GLS: no competing interests to declare; GJP: no competing interests to declare; ETC.: employee of Onyx Pharmaceuticals; JLA: employee of Onyx Pharmaceuticals; CJK: is Vice President, Research, at Onyx Pharmaceuticals; SZ: no competing interests to declare; BACD: no competing interests to declare; WFL: no competing interests to declare; RJS: no competing interests to declare; TDG: no competing interests to declare; GJ: no competing interests to declare.

## Authors’ contributions

GJ was the principle investigator and takes primary responsibility for the paper. SEV, TDG and GJ participated in research design. SEV, MA, DN, JM, ETC. and JLA conducted experiments. JC, MV, GLS, CJK contributed new reagents or analytic tools. SEV, DN, ETC, JLA, and GJ performed data analysis. SEV, YGA, DN, ETC, JLA, SZ, WFL, RJS, TDG and GJ wrote or contributed to the writing of the manuscript. All authors read and approved the final manuscript.

## Supplementary Material

Additional file 1 **Table S1.** Overview of primer and probe sequences for PSMB6, PSMB7, PSMB8, PSMB9, PSMB10 and GUS. Primer design by Vector NTI (Invitrogen) software. **Additional file 1: Figure S1.** Analysis of PSMB5 gene mutations in BTZ-resistant JY cells. Sequencing of PSMB5 gene exon 2 in JY/WT cells and in the BTZ-resistant JY cells: JY/35BTZ and JY/100BTZ. Depicted is the single nucleotide shift (G→T) at nucleotide position 311 in JY/35BTZ and JY/100BTZ along with the corresponding change in a single amino acid substitution (Met45Ile) within the mature PSMB5/β5 protein JY wt: no mutation, JY/35: Met45Ile, JY/100: Met45Ile. **Additional file 1: Figure S2.** Expression of markers involved in antigen presentation in JY/WT and BTZ-resistant sublines. (**A**) HLA-ABC (MHC-I) expression, (**B**) HLA-DR (MHC-II) expression, (**C**) CD80 expression, (**D**) CD86 expression, and (**E**) CD40 expression in JY/WT cells and BTZ-resistant JY cells analyzed by FACS. Results depicted are depicted as MFI over isotype control and represent the mean ± SD of 7- 9 separate experiments. **Additional file 1: Figure S3.** Induction of allogeneic T-cell proliferation by JY/WT and BTZ-resistant JY cells. T cell stimulatory capacity of the short term treatment (24 hours) of BTZ JY/WT (± 10nM BTZ), JY/35 (± 35nM BTZ) and JY/100 (± 100nM BTZ). Results depicted are means ± SD of 4 separate experiments. Click here for file
