# Supplementary material for: Overcoming bortezomib resistance in human B cells by anti-CD20/rituximab-mediated complement-dependent cytotoxicity and epoxyketone-based irreversible proteasome inhibitors
Source: Exp Hematol Oncol. 2013 Jan 10;2:2. doi: 10.1186/2162-3619-2-2 (PMC3560160; doi:10.1186/2162-3619-2-2)
Supplement: Additional file 1 — Table S1. Overview of primer and probe sequences for PSMB6, PSMB7, PSMB8, PSMB9, PSMB10 and GUS. Primer design by Vector NTI (Invitrogen) software. Additional file 1: Figure S1. Analysis of PSMB5 gene mutations in BTZ-resistant JY cells. Sequencing of PSMB5 gene exon 2 in JY/WT cells and in the BTZ-resistant JY cells: JY/35BTZ and JY/100BTZ. Depicted is the single nucleotide shift (G→T) at nucleotide position 311 in JY/35BTZ and JY/100BTZ along with the corresponding change in a single amino acid substitution (Met45Ile) within the mature PSMB5/β5 protein JY wt: no mutation, JY/35: Met45Ile, JY/100: Met45Ile. Additional file 1: Figure S2. Expression of markers involved in antigen presentation in JY/WT and BTZ-resistant sublines. (A) HLA-ABC (MHC-I) expression, (B) HLA-DR (MHC-II) expression, (C) CD80 expression, (D) CD86 expression, and (E) CD40 expression in JY/WT cells and BTZ-resistant JY cells analyzed by FACS. Results depicted are depicted as MFI over isotype control and represent the mean ± SD of 7- 9 separate experiments. Additional file 1: Figure S3. Induction of allogeneic T-cell proliferation by JY/WT and BTZ-resistant JY cells. T cell stimulatory capacity of the short term treatment (24 hours) of BTZ JY/WT (± 10nM BTZ), JY/35 (± 35nM BTZ) and JY/100 (± 100nM BTZ). Results depicted are means ± SD of 4 separate experiments. [file 2162-3619-2-2-S1.pdf]

Additional information to Manuscript 'Overcoming bortezomib resistance in human B cells by antiCD20/rituximab-mediated complement-dependent cytotoxicity and epoxyketone-based irreversible proteasome inhibitors" (Verbrugge *et al*)

Primer name	Type	Concentration	Sequence
PSMB8	forward	300 nM	CCAAGGAATGCAGGCTGTACTA
PSMB8	reverse	300 nM	GAGGCTGCCGACACTGAAAT
PSMB8	Probe	200 nM	TGCGAAATGGAGAACG
PSMB9	forward	300 nM	CTTCACCACAGACGCTATTGCT
PSMB9	reverse	300 nM	ATGACACCCCCGCTTGAG
PSMB9	Probe	200 nM	CCATGAGCCGGGATG
PSMB10	forward	300 nM	CGGTCGTGGCGGACAA
PSMB10	reverse	300 nM	GCCCCACAGCAGTAGATTTTG
PSMB10	Probe	200 nM	CTGCGAGAAGATCCA
GUS	forward	300 nM	GAAAATATGTGGTTG GAGAGCTCATT
GUS	reverse	300 nM	CCGA GTGAAGATCCCCTTTTTA
GUS	probe	200 nM	CCAGCACTCTCGTCGGTGAC TGTTCA

Table S1. Overview of primer and probe sequences for PSMB6, PSMB7, PSMB8, PSMB9, PSMB10 and GUS. Primer design by Vector NTI (Invitrogen) software

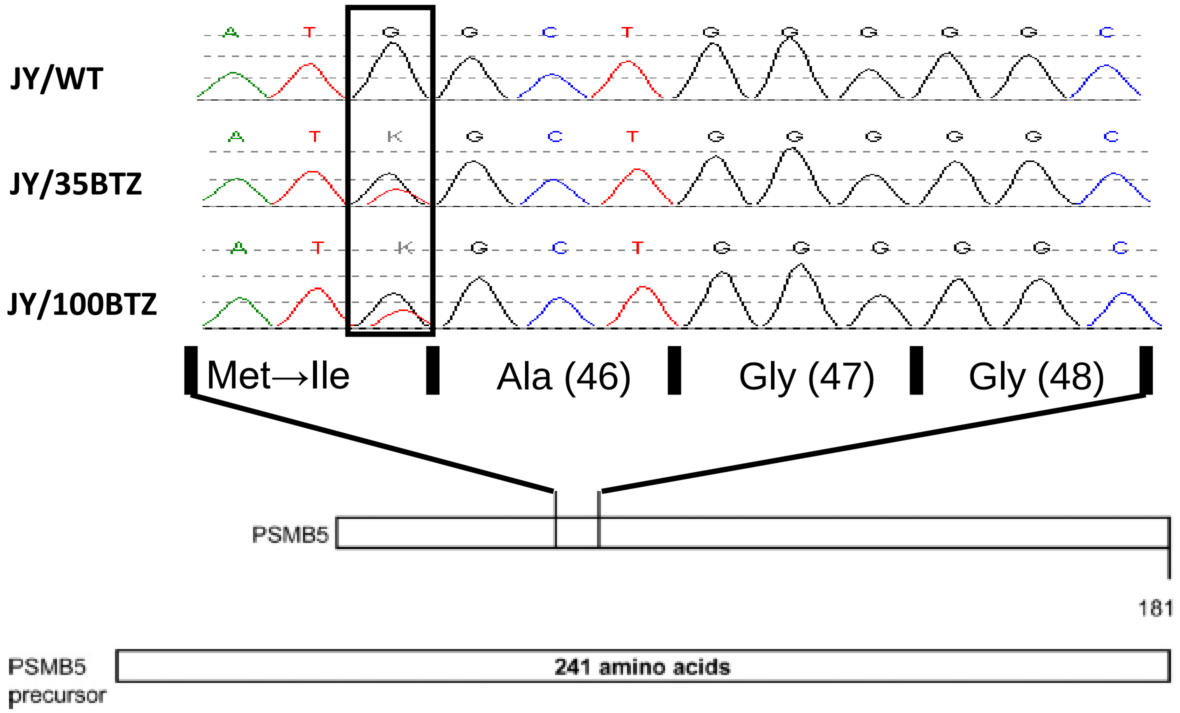

Figure S1. Analysis of PSMB5 gene mutations in BTZ-resistant JY cells. Sequencing of *PSMB5* gene exon 2 in JY/WT cells and in the BTZ-resistant JY cells: JY/35BTZ and JY/100BTZ. Depicted is the single nucleotide shift (G→T) at nucleotide position 311 in JY/35BTZ and JY/100BTZ along with the corresponding change in a single amino acid substitution (Met45Ile) within the mature *PSMB5*/β5 protein JY wt: no mutation, JY/35: Met45Ile, JY/100: Met45Ile

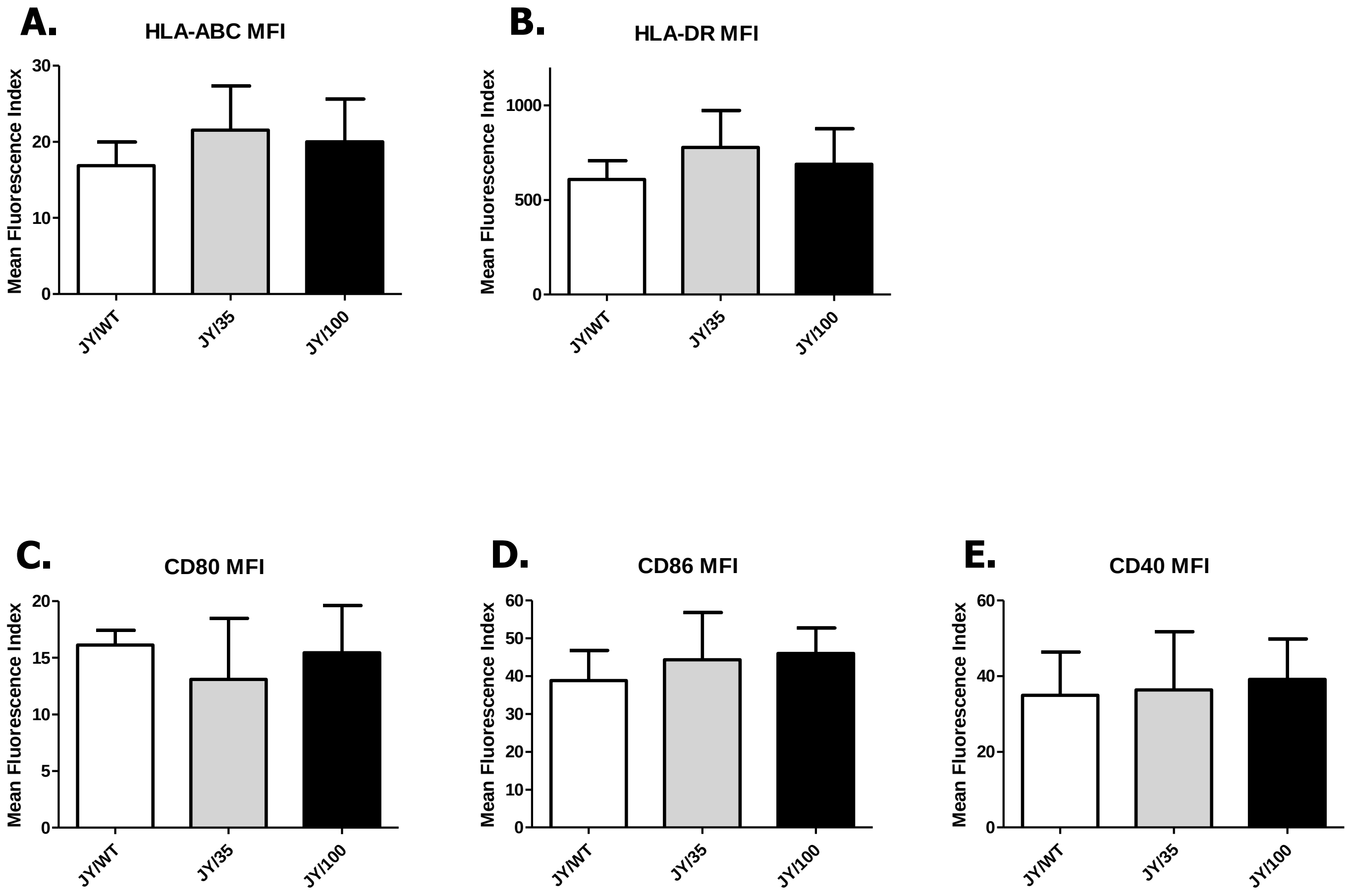

Figure S2. Expression of markers involved in antigen presentation in JY/WT and BTZ-resistant sublines. (**A**) HLA-ABC (MHC-I) expression, (**B**) HLA-DR (MHC-II) expression, (**C**) CD80 expression, (**D**) CD86 expression, and (**E**) CD40 expression in JY/WT cells and BTZ-resistant JY cells analyzed by FACS. Results depicted are depicted as MFI over isotype control and represent the mean ± SD of 7- 9 separate experiments.

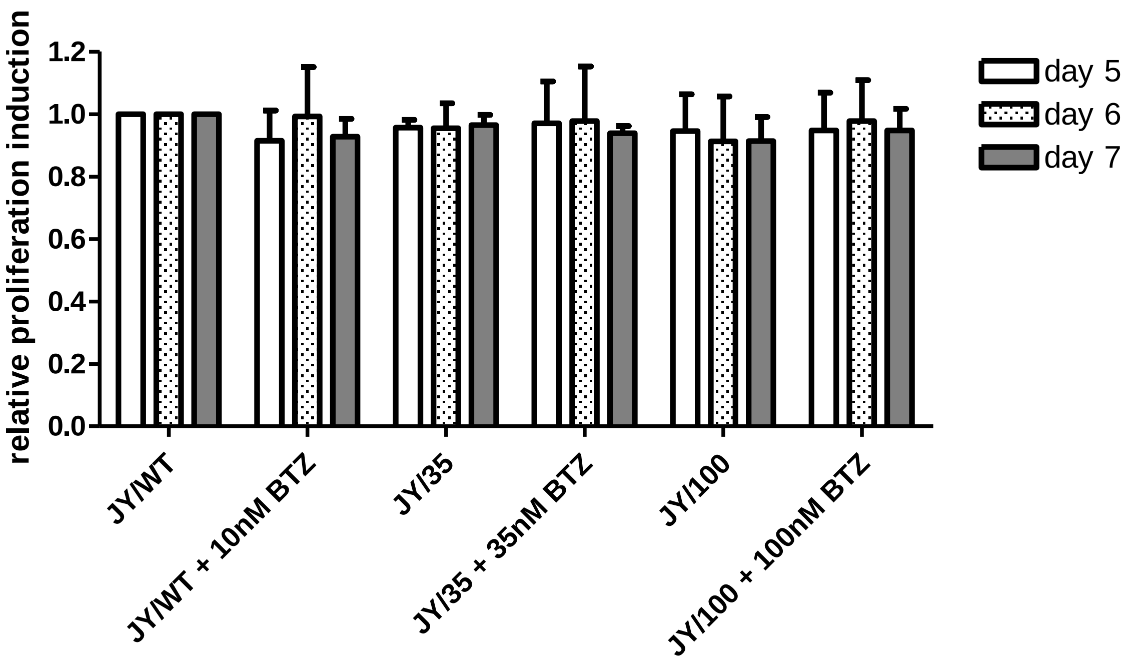

Figure S3. Induction of allogeneic T-cell proliferation by JY/WT and BTZ-resistant JY cells. T cell stimulatory capacity of the short term treatment (24 hours) of BTZ JY/WT (± 10nM BTZ), JY/35 (± 35nM BTZ) and JY/100 (± 100nM BTZ). Results depicted are means ± SD of 4 separate experiments
